# Le traitement conservateur du cancer du sein: expérience d'une équipe tunisienne

**DOI:** 10.11604/pamj.2014.19.148.4195

**Published:** 2014-10-15

**Authors:** Kaouther Dimassi, Anissa Gharsa, Mohammed Badis Chanoufi, Ezzeddine Sfar, Dalenda Chelli

**Affiliations:** 1 Faculté de Médecine de Tunis EL MANAR, Tunisie; 2 Service de Gynécologie-Obstétrique A du centre de Maternité et de Néonatologie, Tunis, Tunisie

**Keywords:** Traitement conservateur, cancer du sein, marges, récidive, XXXXXXXXXX, breast cancer, margin, relaps

## Abstract

En Tunisie, le cancer du sein touche des femmes jeunes avec une taille moyenne au moment du diagnostic à 5 cm. Ces particularités font que la chirurgie radicale reste prédominante. Nous présentons dans ce travail l'expérience de notre équipe en matière de chirurgie conservatrice du cancer du sein. Le but de ce travail est d’évaluer les résultats de ce traitement. Etude rétrospective longitudinale, sur une période de 75 mois. Nous avons inclus toutes les patientes ayant bénéficié d'un traitement conservateur pour une tumeur maligne du sein. Ont été analysés: les caractéristiques épidémiologiques, les aspects radiologiques et histologiques. Le suivi des malades s'est basé sur la détection des récidives. Nous avons évalué le résultat esthétique à la fin de la radiothérapie. Le traitement conservateur a été réalisé dans 23.8% des cas. Le taux de récidives locales était de 6.8% avec une corrélation significative pour une taille tumorale > 30 mm (p= 0.009), l'association d'une composante intracanalaire (p= 0.035), le statut triple négatif (p= 0.003) et des marges d'exérèse < 5mm sans recoupes per-opératoires (p = 0.045). Les facteurs suivants étaient significativement liés au risque de survenue de métastases à distance: le statut triple négatif (p= 0.003), taille tumorale > 30mm (p = 0.006) et l'atteinte ganglionnaire (p = 0.001). Le résultat esthétique était satisfaisant dans 90% des cas. L'augmentation du nombre de patientes pouvant bénéficier d'une chirurgie conservatrice, doit passer impérativement par le développement et la promotion du diagnostic précoce et du dépistage par la mammographie.

## Introduction

Le cancer du sein (CS) est le cancer le plus fréquent chez la femme dans le monde [1] et en Tunisie. Dans notre pays, il y a eu une augmentation de 100% de l'incidence de ce cancer entre 1994 (16/100 000) et 2006 (31.84/100 000) [1] De plus, seulement 32.3% des CS sont diagnostiqués à un stade local et la taille tumorale moyenne au moment du diagnostic est de 5 cm [1] . Enfin, et comparativement aux pays occidentaux, il atteint fréquemment des femmes jeunes. Toutes ces particularités ont fait que la chirurgie radicale est le plus souvent pratiquée pour les patientes tunisiennes. Nous présentons dans ce travail l'expérience de notre équipe en matière de chirurgie conservatrice du cancer du sein. Les buts de ce travail sont: évaluer les indications d'une chirurgie conservatrice pour un cancer du sein et décrire les modalités techniques; étudier l'impact de ce traitement sur la survie sans récidive et sans métastase et déterminer les facteurs pronostiques; évaluer les résultats anatomiques et esthétiques.

## Méthodes

Il s'agit d'une étude rétrospective descriptive longitudinale, menée au service « A » du centre de maternité et de néonatologie de Tunis, sur une période de 75 mois allant de janvier 2007 à mars 2013. Nous avons inclus toutes les patientes ayant bénéficié d'un traitement conservateur (TC) pour une tumeur maligne du sein. Le diagnostic de malignité a été retenu en préopératoire par les prélèvements percutanés ou en per-opératoire par un examen extemporané et confirmé par l'examen anatomopathologique définitif. Pour les lésions infra cliniques, le diagnostic de malignité était retenu après l'examen anatomopathologique de la pièce guidée par Harpon. Les critères d'exclusion étaient les suivants: les patientes ayant eu une tumorectomie initiale dans une autre unité avec un complément de recoupe ou de mastectomie réalisé dans notre service; les échecs du traitement conservateur ayant nécessité un complément de mastectomie dans le même temps opératoire ou dans un intervalle inférieur à 1 mois; les patientes perdues de vue et les patientes ayant des dossiers incomplets (pas de compte rendu anatomopathologique...).

Nous avons analysé les caractéristiques épidémiologiques. Nous avons détaillé l'approche diagnostique en nous basant sur l'examen clinique, l'imagerie et l’étude histologique des microbiopsies. Les images mammographique ont été classées selon le degré de suspicion de malignité selon la classification ACR [2] . Le bilan d'extension comprenait toujours un examen somatique complet, une radiographie du thorax, une échographie hépatique et pelvienne et une scintigraphie osseuse. Nous avons utilisé la classification TNM pour classer les tumeurs avant le traitement (cTNM) et après la chirurgie (pTNM) [3] . Toutes les patientes ont bénéficié d'un TC (tumorectomie et curage ganglionnaire axillaire). L′abord chirurgical était différent selon la localisation de la tumeur. La pièce adressée pour un examen extemporané ou pour un examen anatomopathologique définitif ainsi que les recoupes du lit tumoral étaient toujours orientées avec du fil nylon. L'examen anatomopathologique avait précisé la taille tumorale, ainsi que les éléments du grading de Scarff, Bloom et Richardson (SBR) [4] . De même, l’étude histologique avait précisé la présence éventuelle d'une composante in situ, d'emboles vasculaires, d'un engainement périnerveux et avait détaillé l’état des limites d'exérèse et des recoupes. La marge d'exérèse de sécurité minimale adoptée dans notre étude était de 10 mm. Les résultats de l'expression des récepteurs hormonaux (RH) étaient exprimés en pourcentage et intensité moyenne des noyaux marqués. Les récepteurs hormonaux étaient considérés positifs s'il y avait des récepteurs à l'estrogène ou à la progestérone. Le seuil de positivité était fixé à 10% de cellules marquées (quelle que soit l'intensité du signal). Ces récepteurs étaient considérés négatifs si les deux récepteurs étaient négatifs. Le statut HER2neu ou l'expression de l'oncoprotéine cErbB2 Her2 a été déterminé par méthode immunohistochimique. Un complément de CISH était réalisé pour les tumeurs de score 2 à l’étude immunohistochimique. Les tumeurs HER2 score 2 et 3 avec CISH positive avaient été classées HER2neu positives. Enfin, tous ces paramètres avaient été utilisés pour définir la classe moléculaire intrinsèque de chaque tumeur [5] . Nous avons noté le nombre de ganglions prélevés, le nombre de ganglions atteints et l’éventuelle rupture capsulaire. Les traitements adjuvants ont été détaillés.

Le suivi des malades s'est basé sur la détection des récidives ou des métastases en précisant les délais de survenue de ces complications. Nous avons évalué le résultat esthétique à la fin de la radiothérapie comme suit: **Bon:** bonne cicatrice, pas de déformation du sein ou légère déformation et seins symétriques ou légère asymétrie; **Moyen:** cicatrice visible, déformation moyenne et une asymétrie; Mauvais: mauvaise cicatrice, sein déformé; **Médiocre:** déformation postopératoire majeure du sein et/ou une fibrose rétractile massive de la totalité du sein.

Concernant l’étude statistique, nous avons utilisé le logiciel SPSS version 20 pour Windows. Les courbes de survies ont été calculées à partir de la date du début du traitement selon la méthode actuarielle de Kaplan-Meyer. Pour apprécier cette comparaison, nous avons utilisé le test de Log Rank en conservant le seuil de 5% qui, en matière de statistique médicale, permet de considérer qu'une différence est statistiquement significative (p < 0,05). Les différences entre les pourcentages ont été testées par le test de X2. Enfin, nous avons utilisé le Modèle de Cox pour l’étude multifactorielle des paramètres pronostiques.

## Résultats

**Figure 1:**
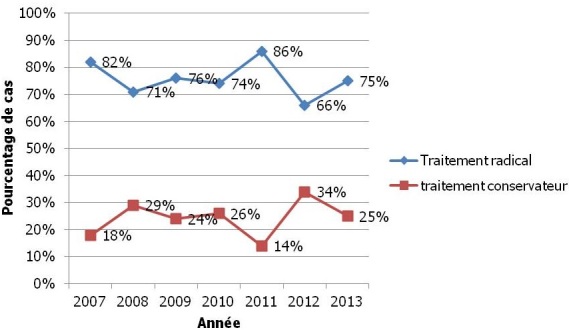
Évolution du taux de traitement conservateur du cancer du sein durant la période de l’étude

Durant la période de notre étude, 485 patientes ont été prises en charge dans notre service pour une tumeur du sein réparties comme suit: 300 tumeurs bénignes (62%) et 185 tumeurs malignes (38%). Parmi les patientes atteintes d'un cancer du sein (CS), 44 ont bénéficié d'une chirurgie conservatrice, soit une fréquence de 23.8%. L’évolution du pourcentage de TC durant la période d’étude est illustrée dans la Figure 1. L’âge moyen des patientes était de 52 ans avec des extrêmes allant de 30 à 75 ans et 36% de patientes âgées de moins de 45 ans. Les patientes ayant eu une ménarche précoce représentaient 38% de l'effectif. Les patientes en activité génitale au moment du diagnostic représentaient 45.5% de la population étudiée. Aucune patiente n'avait reçu un traitement hormonal substitutif de la ménopause. La gestité moyenne était de 3.88 et la parité moyenne de 2.9. L’âge moyen de la première grossesse était de 23.3 ans. La durée moyenne d'allaitement était de 56 mois pour toutes les grossesses obtenues par femme. Le pourcentage des patientes qui n'ont pas utilisé une contraception hormonale était de 73%. Huit de nos patientes (18.2%) avaient au moins un antécédent familial de cancer du sein. Le délai moyen entre l'apparition des symptômes et la 1ière consultation était de 3.3 mois avec des extrêmes allant de 7 jours à 18 mois. La découverte d'un nodule mammaire par l'autopalpation était le motif de consultation de 68% de l'effectif. Un dépistage systématique par mammographie, demandé à l'occasion d'une consultation en gynécologie, a concerné une seule patiente (2.2%). La taille clinique moyenne des lésions était de 30.2 mm. L’évaluation statistique n'a pas montré de corrélation entre l’âge de découverte de la tumeur et la taille clinique (p= 0.541). De même, Nous n'avons pas pu établir une corrélation entre la taille clinique et le délai entre le premier symptôme et la 1ère consultation (p= 0.445). Les tumeurs avaient touché les quadrants supérieurs dans 70% des cas. Les lésions ont intéressé le sein droit dans 53.2% de l'effectif (25 cas) et dans 46.8% des cas le sein gauche. Nous avons objectivé la présence d'adénopathies axillaires homolatérales chez 11 patientes, soit 25% des cas. Toutes les lésions axillaires ont été classées stade N1. La répartition des tumeurs selon la classification T.N.M est résumée dans le Tableau 1.

**Tableau 1:** Répartition des patientes selon la classification TNM

Classification clinique pré-thérapeutique (cTNM)	Nombre de lésions	Pourcentage
T0N0Mx	3	6.4%
T1N0Mx	13	27.6%
T1N1Mx	8	17%
T2N0Mx	18	38.3%
T2N1Mx	5	10.7
Total	47	100%

**Figure 2:**
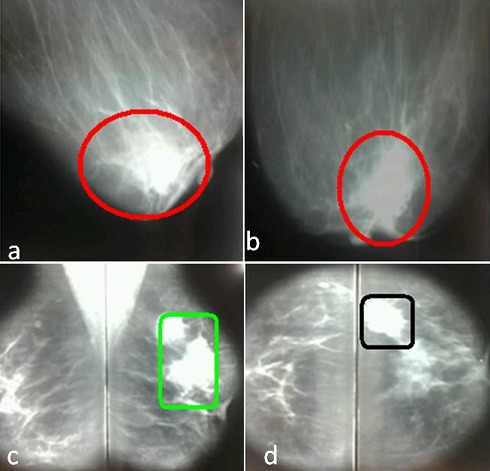
Aspects mammographiques des patientes de l’étude. (a et b): opacité spiculée à centre dense, mal limitée classée ACR5; (c et d): lésions classées ACR4

**Figure 3:**
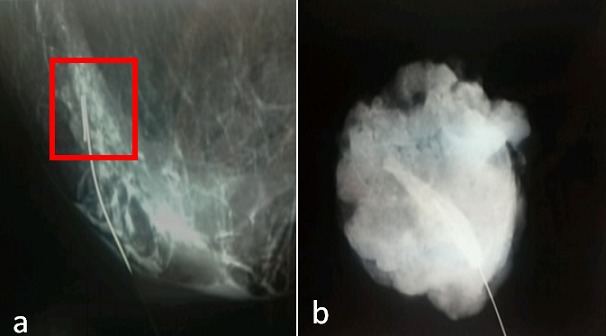
(a): radiographie de la lésion repérée par Harpon; (b): radiographie de la pièce après tumorectomie

La mammographie a été pratiquée chez 41 patientes (93.2%), couplée à une échographie mammaire dans 48% des cas et 3 patientes ont seulement eu une échographie mammaire. Nous avons trouvé 47 lésions dont 66% ont été classées ACR5 et 27.6% classées ACR4 (Figure 2) . Les lésions étaient bilatérales dans trois cas, associant ACR 3 et 5 dans deux cas et dans un autre cas, il s'agissait d'ACR 4 et 5. La taille moyenne des lésions retenue par le couple échographie-mammographie était de 26.75 mm. La concordance de taille entre examen clinique examen radiologique était de 87%. Le diagnostic histologique a été posé en préopératoire dans 30 cas (63,82%). Ainsi, une microbiopsie a été indiquée pour 29 lésions cliniquement palpables (61.7%). Une ponction associée à une biopsie avait été pratiquée chez une seule patiente. Ailleurs, et en présence d'une lésion infra clinique (3 cas), le diagnostic était obtenu par biopsie chirurgicale après repérage par harpon (Figure 3). Le diagnostic était obtenu par un examen extemporané fait dans le même temps opératoire dans 14 cas (29.7%). A noter que le bilan d'extension, associant un examen clinique complet, une radiographie du thorax, une scintigraphie osseuse, une échographie pelvienne et hépatique, était négatif dans tous les cas. Aux termes de ces explorations, nous avons classé les tumeurs selon l'Union Internationale Contre le Cancer (UICC) [3] en: Stade 0: 3 cas; Stade I: 13 cas; Stade IIA: 26 cas; Stade IIB: 5 cas.

Lors de l'examen histologique définitif, nous avons recensé 84.6% de carcinomes canalaires infiltrants (CCI). La taille tumorale histologique moyenne était de 23,47mm. Les tumeurs ayant une taille inférieure ou égale à 30mm représentaient 79.54% de l'ensemble des tumeurs (35 cas). Les tumeurs classées II ou III selon le grade SBR représentaient 90% des cas. Nous n'avons pas trouvé de corrélation entre la taille clinique et le score SBR (p= 0.331). La présence d'emboles tumoraux a été confirmée dans 8 cas (21.6%). Un engainement périnerveux était trouvé chez 4 patientes. Une composante intracanalaire (Carcinome In Situ non étendu) était associée à 17 cas de CCI (31.2%). Une recherche des RH était réalisée chez 31 patientes (70.45%). Elle était positive chez 58% des patientes (18 cas). L’étude immunohistochimique de l'expression du marqueur cellulaire HER2neu a été réalisée chez 30 patientes (68%). Elle était positive chez deux parmi elle, soit une fréquence de 6.66%. Les tumeurs triples négatives ont été la classe prédominante dans notre série (22.7%). La répartition des tumeurs selon leur classification moléculaire intrinsèque est résumée dans le Tableau 2. Nous avons pu récupérer 27 résultats sur l'expression de KI67. Elle était positive chez 9 patientes (33.3%). Le taux moyen de positivité était de 49%. Nous avons enlevé 682 ganglions, soit une moyenne de 16.2 ganglions par patientes; seules 3 patientes avaient un curage ramenant moins de 10 ganglions. Nous avons récupéré 72 ganglions pathologiques chez 20 patientes. Le nombre moyen de ganglions envahis était de 3.6. Une rupture capsulaire a été trouvée chez 9 patientes.

**Tableau 2:** Répartition des tumeurs selon leur classification moléculaire intrinsèque

	Luminal A (RH + faible, HER2-)	Luminal B (RH+, HER2 -)	Her2 neu + (RH-, surexpression de HER2)	Basal-like: Triples négatives (RH-,HER2 -)	Indéterminé
Nombre de cas	7	9	2	10	16
Pourcentage	16%	20.5%	4.5%	22.7%	36.3%

**Figure 4:**
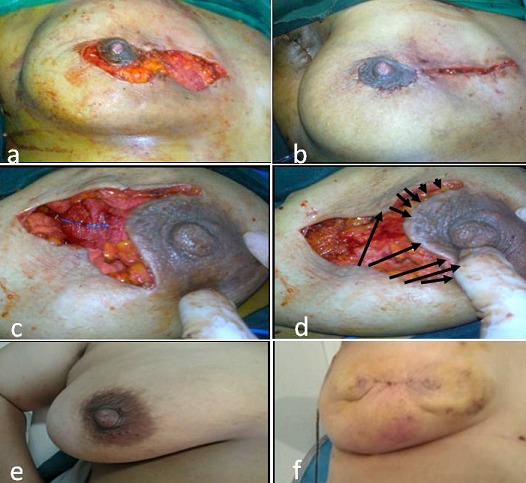
(a): remodelage glandulaire après tumorectomie; (b): cicatrice après recentrage de la plaque aréolomamelonnaire et fermeture cutanée; (c): incision, tumerectomie et remodelage; (d): schématisation de la fermeture cutanée

Concernant la technique chirurgicale, l′abord chirurgical était différent selon la localisation de la tumeur. Toutes les patientes ont eu une tumorectomie large selon le siège de la tumeur avec un remodelage glandulaire classique. Aucune patiente n'a bénéficié d'une plastie controlatérale dans le même temps opératoire. La Figure 4 illustre quelques techniques de tumorectomie- remodelage réalisées dans notre service. Parmi nos patientes, une avait présenté une tumeur du sillon sous mammaire, nous avons procédé par une incision transversale ellipsoïde centrée par la tumeur. La résection était large, jusqu'au plan pectoral, emmenant la tumeur. Chez une seule patiente, la tumeur avait touché la plaque aréolo mamelonnaire. La tumeur avait mesuré 35*25 mm et était à 10 mm du mamelon. Chez cette patiente, nous avons opté pour une pamectomie par technique transversale. La tumorectomie centrale est allée jusqu'au plan pectoral avec un remodelage glandulaire identique de la profondeur vers la superficie. La fermeture cutanée était directe et la cicatrice était horizontale (Figure 4e, f). L'examen extemporané a conclu des berges saines dans 52.3% des cas (23 cas) dont cinq avaient des marges de sécurité inférieure à 10 mm et seulement trois d'entre elles avaient eu des recoupes du lit tumoral. Les patientes ayant des berges envahies (45.5%) ont bénéficié de recoupes systématiques du lit tumoral dans le même temps opératoire. Toutes les recoupes étaient saines. La présence d'une masse spiculée à la mammographie et le grade SBRIII avaient une corrélation significative avec la positivité des berges d'exérèse (p respectivement à 0.001 et 0.036). Par contre, les microcalcifications, la présence d'emboles tumoraux, la composante intracanalaire associée, l’âge inférieur à 40 ans et une taille mammographique supérieure à 3cm n’étaient pas des facteurs de positivité des berges d'exérèse dans notre série.

Aucune de nos patientes n'avait reçu un traitement néo adjuvant de réduction tumorale avant la chirurgie. Les patientes qui ont bénéficié d'une radiothérapie adjuvante représentaient 95.45% de l'effectif. Le délai entre la chirurgie et la radiothérapie variait de 1 à 10 mois avec une moyenne de 3 mois et demi. Quarante et une patientes ont bénéficié d'une radiothérapie à la dose de 50 Gy sur la paroi thoracique et le sein en 5 semaines environ, par fraction 2 Gy 5 fois/semaine. Un complément d'irradiation sur le lit tumoral a été délivré pour 21 patientes (50%) à la dose de 14 Gy. La chimiothérapie adjuvante a été réalisée dans 68.2% des cas. Toutes les patientes ayant des récepteurs à l'oestrogène positifs ont bénéficié d'un complément d'hormonothérapie, soit 17 patientes (40.9%). Une seule patiente a bénéficié d'un traitement à base d'Herceptine. Sur les 44 patientes opérées, nous avons noté 3 cas de récidive locale, soit une fréquence de 6.8%. L’âge moyen au moment de la récidive était de 43 ans. Nous n'avons pas objectivé de relation significative entre le risque de récidive locale et les caractéristiques suivantes: âge de la patiente (p = 0.09), la présence d'emboles tumoraux (p= 0.07). Par ailleurs, une relation significative était objectivée pour une taille supérieure à 30 mm (p= 0.009), l'association d'une composante intracanalaire (p= 0.035), le statut triple négatif (p= 0.003) et des marges d'exérèse inférieure à 5mm sans recoupes per-opératoires (p = 0.045). Trois patientes avaient présenté des métastases osseuses, soit une fréquence de 6.8%. Les délais d'apparition étaient respectivement de 7, 11 et 21 mois. Les facteurs suivants étaient significativement liés au risque de survenue de métastases à distance: le statut triple négatif (p= 0.003), taille tumorale clinique supérieure à 30mm (p = 0.006) et l'atteinte ganglionnaire (p = 0.001). Par ailleurs, nous n'avons pas pu établir une relation significative entre la rechute locale et la survenue de métastase (p= 0.6).

**Figure 5:**
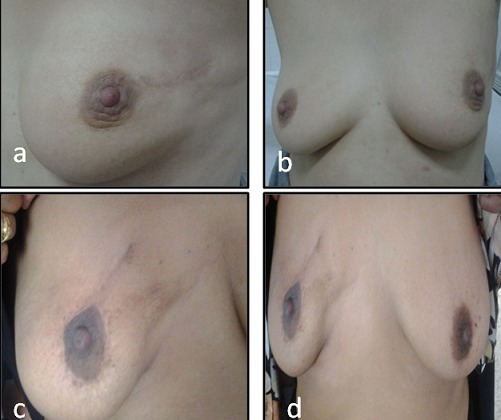
Résultats esthétiques à 2 ans du traitement chirurgical. (a): résultats esthétiques d'une tumorectomie du quadrant supéro-externe gauche; (b): symétrie des deux seins; (c): cicatrice de tumorectomie de quadrant supéro-interne droit; (d): symétrie des seins

Nous avons évalué les résultats esthétiques pour quelques patientes par téléphone et pour d'autres par un examen à la consultation. Les patientes qui ont jugé le résultat « bon » ou « moyen » (selon les critères suivants: degré de symétrie des seins, déformation du sein traité et qualité de la cicatrice) représentaient 90% des cas. La conservation de la symétrie des seins a été retrouvée chez 72.1% des femmes et 41.9% des cas ont rapporté une diminution du volume mammaire. L'anxiété, rapportée par 27.9% des patientes vis-à-vis du sein traité, était associée à une crainte de récidive de la maladie. La Figure 5 illustre les résultats esthétiques chez deux patientes de notre étude.

## Discussion

En Tunisie, le CS est le premier cancer de la femme. De plus, sa fréquence est en augmentation régulière. Ainsi l'incidence standardisée sur l’âge du CS en Tunisie est passée de 16.7 pour 100000 femmes en 1994 à 31.84/100 000 en 2006 [1] . Une des particularités du CS en Tunisie est sa survenue chez les femmes jeunes. En effet, la moyenne d’âge se situe autour de la cinquantaine dans la majorité des séries tunisiennes. Dans notre série, l’âge moyen était de 52 ans. De plus, toutes les études d'incidence sont en faveur d'une augmentation du cancer du sein dans la tranche d’âge inférieure à 40 ans en Tunisie comme en Afrique du nord. En France, ce pourcentage ne dépasse pas 5% [6] . Cette particularité épidémiologique de la population concernée par le CS dans notre pays représente un argument en faveur de la diffusion d'une chirurgie conservatrice et de l'oncoplastie.

Le bien fondé des traitements conservateurs (TC) dans la prise en charge des cancers du sein de moins de 3cm n'est plus à démontrer. En Tunisie, à l'institut Salah Azaiez (centre de référence régional de l'Organisation mondiale de la santé (OMS) pour les CS et du col), ce taux est passé de 4% en 1987, 5% en 1996 à 22% en 2000. Dans notre série, en 2009, 24% des patientes ont bénéficié d'un TC et en 2012 ce taux est passé à 33%. Cette augmentation s'explique par l'introduction progressive du TC dans nos moeurs thérapeutiques et la diffusion progressive des programmes de dépistage. Cependant, cette pathologie continue à être diagnostiquée à un stade avancé dans notre pays avec une taille moyenne au moment du diagnostic à 5cm [1] . Ainsi, en Tunisie, le nodule mammaire demeure le motif de consultation le plus fréquent (66% à 95%). Ce nodule mammaire a été découvert par autopalpation chez 68% de nos patientes. Une seule patiente a été diagnostiquée au stade infraclinique c′est-à-dire lors d'une mammographie systématique demandée en consultation. Alors que dans les séries européennes, le CS peut être dépisté dans prés des 40% des cas [7] . Ce ci s'explique pas l'absence à ce jour d'un programme de dépistage organisé du CS en Tunisie. En effet, les prescriptions de mammographies systématiques sont soit individuelles ou encore s'intègrent dans des programmes de dépistage régionaux de durée limitée. Enfin, tous les éléments suscités contribuent à retarder la généralisation du TC comparativement aux séries occidentales où les pourcentages atteignent facilement les 60 à 75%. [8] .

### Indications du traitement conservateur

Dans notre série, nous avons réalisé un TC pour des tumeurs unifocales de taille inférieure à 5 cm. En effet, l’équivalence, en termes de survie, entre le TC et la mastectomie est formellement démontrée pour les tumeurs allant jusqu’à 4 cm et 5 cm [9] . Les tumeurs centrales superficielles rétroaréolaires étaient classiquement traitées par mammectomie. Actuellement, la conservation du sein dans les tumeurs rétroaréolaires est rendue possible grâce aux techniques dérivées de la chirurgie plastique impliquant l'ablation de la plaque aréolomamelonnaire (PAM) [10] . Dans notre série, nous avons pratiqué une pamectomie devant une lésion centrale superficielle. Cette patiente n'a pas présenté de récidive locale. Les taux de récidive locale des différentes études en cas de TC sur des tumeurs centrales varie de 0 à 40% [10] . Concernant les carcinomes canalaires in situ (CCIS), la mastectomie totale a toujours été considérée comme traitement de référence garantissant le contrôle local da la maladie dans 98% des cas [11] . D'autant plus que, la chirurgie conservatrice dans ce contexte est associée à des taux de récidive locale de 7 à 10% à cinq ans et de 30 à 35% à dix ans [11, 12] Cependant, actuellement, la meilleure connaissance des facteurs pronostiques de la récidive locale permet de proposer une stratégie conservatrice à un grand nombre de patientes sans influencer négativement leur pronostic [13] .

### Technique utilisée pour la tumorectomie: Chirurgie conservatrice unilatérale ou « technique classique »

C'est la technique adoptée dans notre série. Elle implique l'exérèse au large de la tumeur avec un remodelage glandulaire systématique. La confection de deux lambeaux de glissement permet de remodeler la glande pour la majorité des tumorectomies. Pour des exérèses plus larges, ou des tumeurs situées dans des zones à haut risque de déformation (quadrants inférieurs), il faut faire appel à un lambeau glandulaire de rotation, voire à un recentrage de la PAM. La préparation du lambeau glandulaire répond aux mêmes principes que ceux du remodelage glandulaire simple. Un des lambeaux est sectionné transversalement et mobilisé par rotation, parfois par avancement, avant d’être fixé dans la cavité de tumorectomie. Lors d'exérèses importantes, l'aréole peut être déviée vers le lit tumoral. Il faut la repositionner au sommet du dôme mammaire [14] . Lorsque ces techniques sont insuffisantes, il faut faire appel aux techniques oncoplastiques qui nécessitent toujours une plastie de symétrisation. Nous n'y avons pas eu recours.

### Limites d'exérèse: marges et berges d'exérèse

L'obtention de berges d'exérèse saines est un impératif constituant un facteur de risque de récidive locale indépendant et contrôlable par la qualité de la chirurgie [15–17] . En revanche, il n'existe pas de consensus histologique en termes de marge d'exérèse (distance en millimètre entre les limites tumorales et les berges d'exérèse) pour considérer que la taille de la pièce de tumorectomie est suffisante. Ainsi, dans l’étude présentée par AZU et al [18] , 11% des chirurgiens ont défini des marges saines par l'absence de cellules tumorales au niveau des berges colorées, 42% les définissent à 1-2 mm, 28% les limitent à plus de 5mm et 19% à 10 mm. Ce qui est actuellement retenu aux USA c'est l'impératif d'avoir des berges saines [16] . Ce ci dit, il est admis que le taux de reliquats tumoraux en cas de berges envahies se situe entre 29 et 70% [19] . En cas de berges saines mais de marges de sécurité insuffisantes ce taux se situe entre 0 et 36% [19] . Enfin, le taux de récidives locales est retenu plus important lorsque les marges minimales sont inférieures à 2 mm [19] . Dans ce travail, nous avons objectivé une relation significative entre des marges saines inférieures à 5 mm et la survenue de récidive locale (p= 0.045). Ainsi, nous avons considéré 10mm comme distance minimale pour parler de marges saines. En per-opératoire et lors de l'examen extemporané, 45% des patientes avaient des berges envahies et avaient eu des recoupes systématiques du lit tumoral dans le même temps opératoire. Dans la littérature, le taux de reprise des berges après tumorectomie varie entre 17 et 59% [20] . Les données de l’étude coréenne de Bulent K [21] révèlent que l'atteinte des ganglions axillaires, la présence d'emboles vasculaires et de composante intra canalaire extensive ainsi que les masses spiculées et les microcalcifications à la mammographie sont des facteurs de positivité des berges d'exérèse après un TC. De même dans notre étude nous avons retrouvé cette relation significative pour l'aspect spiculé de la tumeur ainsi que pour le grade SBR III.

Concernant l'influence du type moléculaire des lésions sur la positivité des berges d'exérèse, peu d’études objectivent une relation significative. Ainsi, Sioshani et al [22] rapportent que les triples négatifs sont associés à un risque significativement accru de marges envahies alors que Ataly et al. [23] objectivent ce même risque pour les tumeurs HER2 neu +. Ailleurs, la taille tumorale apparait comme un facteur de risque de positivité des berges d'exérèse [24] . Il faut noter qu'en per opératoire, la détermination par le chirurgien des limites de l'exérèse adéquates est parfois difficile à définir surtout en cas de lésion infraclinique. Ceci peut constituer un obstacle à la réalisation d'un geste complet d'emblée. Il est souvent proposé une reprise des berges de la tumorectomie guidée par les données histologiques de la pièce opératoire lorsque la distance entre le cancer et les marges d'exérèse sont inférieures à 2-3 mm [25] . La réalisation de cette reprise chirurgicale diminue le risque de récidive locale [15] . Certaines équipes proposent la réalisation de recoupes systématiques au niveau de la loge d'exérèse lorsqu'un traitement chirurgical conservateur est entrepris [15, 26] . En effet, L’étude histologique des berges de tumorectomie est souvent délicate. Il existe un risque non négligeable de faux positif ou de faux négatif lié à la friabilité des bords de la tumorectomie et à l'aspect déformable du tissu mammaire prélevé. Ainsi, 45 à 70% des recoupes réalisées pour berges de tumorectomie non in sano ne mettent pas en évidence de lésions tumorales résiduelles [27] . Il en est de même lorsque l'on étudie les recoupes effectuées de façon systématique lors de la tumorectomie. Le pourcentage de cas de tumorectomies non in sano ayant des recoupes saines varie entre 11 et 40% [26, 28] . Dans notre série toutes les recoups étaient saines. Lorsque les recoupes ne présentent pas d'atteinte carcinomateuse alors que les berges de la tumorectomie sont atteintes, on peut considérer que la marge d'exérèse de sécurité est suffisante et sursoir à une reprise chirurgicale. Ainsi, le taux de ré-intervention lorsque des recoupes sont effectuées lors du traitement chirurgical initial est plus faible que lorsque seule une tumorectomie est réalisée [15, 29–31] . Ainsi, la réalisation de recoupes systématiques et l’étude histologique de ces dernières permettent d’éviter une reprise chirurgicale dans 19 à 59% des cas [26, 28, 30, 32] . De plus, les recoupes majorent les marges de sécurité et permettent dans certains cas de mettre en évidence une multicentricité méconnue. Dans 2-10% des tumorectomies avec des marges d'exérèse saines, il est retrouvé au sein des recoupes des lésions de carcinome in situ ou invasif [26, 28] . La réalisation de recoupes lors de la tumorectomie constitue donc selon les auteurs une aide à la détection de cancer résiduel ou d'une atteinte plurifocale passée initialement inaperçue et, de ce fait, elle constitue un facteur pronostique indépendant. L'atteinte des recoupes semble être plus prédictive de la présence de maladie résiduelle que l'atteinte des berges. Tous ces arguments avaient motivé la réalisation des recoupes systématiques dans cette série.

### Chirurgie axillaire

Dans notre série, la conduite a été de réaliser systématiquement un curage axillaire. Nous avons réséqué une moyenne 16.2 ganglions par patiente avec des extrêmes allant de 4 à 34 ganglions. Seules 3 patientes avaient un curage ramenant moins de 10 ganglions. Ces résultats sont concordants aux différentes séries tunisiennes. Depuis des années, et afin de diminuer la morbidité du curage axillaire classique, le concept de ganglion sentinelle a fait peu à peu son apparition. Dans une population de patientes atteintes d'un petit cancer du sein, dont le taux de risque d'atteinte ganglionnaire est de 30% environ, l'intérêt premier de cette technique est d’éviter un curage axillaire aux 70% des patientes dont l'aisselle est indemne. Dans des mains expérimentées, le taux de détection du ganglion sentinelle est actuellement de 95% [33] . Nous n'avons pas pu réaliser cette technique vu que nous ne disposons pas du matériel nécessaire pour la détection du ganglion radioactif. Seul l'institut Salah Azaiez dispose de cette technique dans notre pays. Plusieurs essais ont montré que les taux de conservation mammaire pouvaient être augmentés grâce aux traitements néo adjuvants (chimiothérapie, radiothérapie voire hormonothérapie) [34] . Aucune de nos patientes n'a pu bénéficier de cette conduite et il serait effectivement intéressant d'introduire ce principe dans nos pratiques afin d'augmenter le taux de conservation mammaire chez nos patientes jeunes et avec des tumeurs plutôt volumineuses au moment du diagnostic.

### Résultats du traitement conservateur

Le taux de rechute locale, après un traitement radio-chirurgical conservateur d'un CS invasif, varie entre 4 et 20% à cinq ans selon les séries [26] . Ce taux ne dépasse pas les 2% dans les séries les plus récentes avec des patientes bien sélectionnées et une radiothérapie Boost sur le lit tumoral [35] . Dans notre série, ce taux était de 6.8%. Les deux conséquences principales de la rechute tumorale locale après TC sont d'une part, d'ordre psychologique et d'autre part, pronostique. Ce risque de récidive mammaire diminue régulièrement à mesure que l’âge augmente [35, 36] . Nous n'avons pas pu mettre en évidence de relation significative entre l’âge jeune et le risque de récidive locale (p = 0.009).

Dans notre série, La taille tumorale moyenne était de 35mm pour les patientes ayant des récidives. Une relation significative (p= 0.009) était trouvée pour une taille supérieure 30 mm. Ce facteur a fait l'objet de controverses [37] . La positivité des berges d'exérèses est aussi un important facteur de risque de récidive locale en cas de TC [36] . La présence de composante intracanalaire associée ou un CIS ont été associés significativement à la récidive locale dans notre étude. Ailleurs, les résultats sont hétérogènes et il est admis que ce facteur perd son influence en cas de résection complète avec des berges saines [36] . La présence d'emboles tumoraux intra vasculaires (sanguins ou lymphatiques) dans la tumeur est un facteur de risque de récidive locale. Ce risque est de 25% à dix ans, contre 8% en absence d'emboles [36] . Dans notre série, la présence d'emboles tumoraux n'avait pas de relation significative vu la taille de l’échantillon et la durée courte de suivi (p= 0.07). Dans d'autres séries tunisiennes, la présence d'emboles vasculaires était un facteur significatif pour la survie sans récidive à 5 ans (p = 0.013). Le statut HER2+ a d'abord été rapporté comme facteur pronostique général indépendant avant d’être considéré comme un facteur prédictif pour la réponse au trastuzumab en situation métastatique puis adjuvante. Dans une étude sur 793 patientes (n'ayant pas reçu de trastuzumab) traitées entre 1998 et 2001, le taux actuariel de rechute locale après un traitement locorégional optimal (97% de berges saines) était de 1,8% à cinq ans. Avec une durée médiane de suivi de 70 mois, les taux de rechute étaient, respectivement de 0.8, 1.5, 8.4 et 7.1% pour les lésions classées en luminal A, luminal B; un statut HER2+ et triple négatives [36] . Dans notre étude, L'effet du statut triple négatif avait une relation significative avec la survenue de récidive locale (p= 0.003). L'envahissement ganglionnaire axillaire reste de loin le principal facteur de risque de récidive locorégionale [36, 38] . Plusieurs études ont décrit un « nodal ratio », correspondant au rapport (en%) entre nombre de ganglions envahis et prélevés, qui serait peut-être plus « fiable » dans la prédiction du risque de récidive locorégional, avec des seuils cependant variables et compris entre 20 et 25% [39, 40] . L'impact de la radiothérapie est plus important, tant en termes de contrôle local que de survie spécifique, en cas d'envahissement ganglionnaire (pN +) [41] .

### Résultats esthétiques

Dans 20 à 30% des cas, le TC s'accompagne de séquelles liées à la chirurgie et à l'irradiation [8] . Les séquelles consistent essentiellement en une asymétrie de volume ou une asymétrie de position de l'aréole (41.9% des cas dans notre série); une déformation du sein par perte de substance glandulaire ou rétraction cutanée (9.4% de nos cas); une cicatrice élargie, rétractile et inesthétique ou encore une bride cicatricielle. L’évaluation des résultats esthétiques peut être standardisée par l'utilisation de classifications des séquelles esthétiques du TC. [8] . Ces classifications sont proposées comme guide pour aider les chirurgiens à planifier la reconstruction du sein.

## Conclusion

Malgré une légère augmentation du taux de chirurgie conservatrice enregistré ces dernières années, ce taux reste encore faible dans notre pays avec cependant des taux de survie et de récidive locorégionale comparables aux données de la littérature. L'augmentation du nombre de patientes pouvant bénéficier d'une chirurgie conservatrice, doit passer impérativement par le développement et la promotion du diagnostic précoce et du dépistage par la mammographie. L'information et l’éducation sanitaire du public ainsi que la sensibilisation des professionnels de la santé sont autant d'actions indispensables afin d'atteindre de tels objectifs.
